# Bis(3-nitro­anilinium) sulfate

**DOI:** 10.1107/S1600536809013762

**Published:** 2009-04-22

**Authors:** Lingling Feng, Yubang Hou, Xuejian Yong, Feng Bao

**Affiliations:** a Department of Chemistry, Central China Normal University, Wuhan 430079, People’s Republic of China

## Abstract

In the title salt, 2C_6_H_7_N_2_O_2_^+^·SO_4_^2−^, all the non-H atoms of both cations and the S atom and two O atoms of the anion lie on a crystallographic mirror plane. In the crystal structure, N—H⋯O and C—H⋯O hydrogen bonds help to establish the packing.

## Related literature

For a related structure, see: Bao *et al.* (2006 ▶). For background, see: Barclay & Hoskins (1965 ▶); Elmali *et al.* (1997 ▶); Tahir *et al.* (1996 ▶).
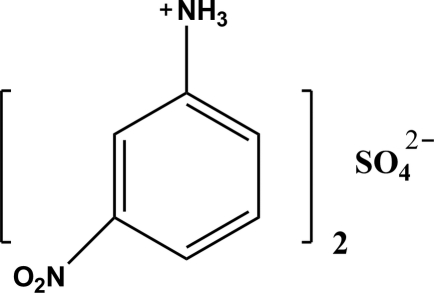


## Experimental

### 

#### Crystal data

2C_6_H_7_N_2_O_2_^+^·SO_4_^2−^
*M* *_r_* = 374.33
Orthorhombic, 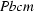

*a* = 7.9177 (16) Å
*b* = 30.843 (6) Å
*c* = 6.3924 (13) Å
*V* = 1561.1 (5) Å^3^
*Z* = 4
Mo *K*α radiation
μ = 0.26 mm^−1^
*T* = 290 K
0.12 × 0.10 × 0.08 mm

#### Data collection

Bruker SMART CCD diffractometer
Absorption correction: multi-scan (*SADABS*; Sheldrick, 1997 ▶) *T*_min_ = 0.959, *T*_max_ = 0.979
12364 measured reflections
1845 independent reflections
1735 reflections with *I* > 2σ(*I*)
*R*_int_ = 0.027

#### Refinement

*R*[*F* ^2^ > 2σ(*F* ^2^)] = 0.061
*wR*(*F* ^2^) = 0.144
*S* = 1.17
1845 reflections
148 parameters
H-atom parameters constrained
Δρ_max_ = 0.36 e Å^−3^
Δρ_min_ = −0.44 e Å^−3^

### 

Data collection: *SMART* (Bruker, 2001 ▶); cell refinement: *SAINT-Plus* (Bruker, 2001 ▶); data reduction: *SAINT-Plus*; program(s) used to solve structure: *SHELXS97* (Sheldrick, 2008 ▶); program(s) used to refine structure: *SHELXL97* (Sheldrick, 2008 ▶); molecular graphics: *PLATON* (Spek, 2009 ▶); software used to prepare material for publication: *PLATON*.

## Supplementary Material

Crystal structure: contains datablocks I, global. DOI: 10.1107/S1600536809013762/hb2912sup1.cif

Structure factors: contains datablocks I. DOI: 10.1107/S1600536809013762/hb2912Isup2.hkl

Additional supplementary materials:  crystallographic information; 3D view; checkCIF report

## Figures and Tables

**Table 1:** Hydrogen-bond geometry (Å, °)

*D*—H⋯*A*	*D*—H	H⋯*A*	*D*⋯*A*	*D*—H⋯*A*
N1—H1*A*⋯O6^i^	0.89	2.37	3.226 (4)	161
N1—H1*B*⋯O5	0.89	2.30	2.987 (4)	134
N1—H1*B*⋯O5^ii^	0.89	2.39	2.928 (3)	119
N3—H3*A*⋯O7^iii^	0.89	1.86	2.755 (4)	176
N3—H3*B*⋯O5^iv^	0.94	1.84	2.756 (3)	164
C4—H4⋯O4^v^	0.93	2.48	3.229 (5)	138
C6—H6⋯O6^i^	0.93	2.29	3.139 (4)	151
C8—H8⋯O3^vi^	0.93	2.45	3.165 (5)	133
C10—H10⋯O2^vii^	0.93	2.51	3.184 (5)	130

Symmetry codes: (i) 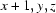; (ii) 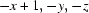; (iii) 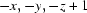; (iv) 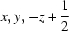; (v) 
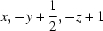
; (vi) 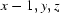; (vii) 
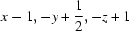
.

**Fig. 1.**
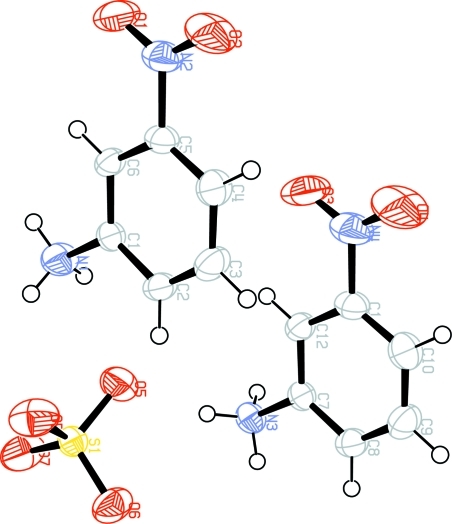
Vew of (I), with displacement ellipsoids drawn at the 50% probability level. H atoms are drawn as spheres of arbitrary radius and the hydrogen bond is indicated by a double-dashed line.

## supplementary crystallographic information

### Comment

The Schiff base that is derived by condensing acetylacetone and a substituted aniline rearranges itself upon being deprotonated in order to chelate to copper (Barclay & Hoskins, 1965; Elmali *et al.*, 1997; Tahir *et al.*, 1996). In our hands, in the reaction of the 3-nitro substituted ligand with copper sulfate, the ligand is cleaved (probably to starting reactants). A analogous structure had been reported by our group (Bao *et al.*, 2006).

In the title compound, (I), the asymmetric unit consists of half sulfate anion and two halves of 3-nitroaniline cations (Fig. 1). H1B, H3B and O5 atoms were symmetry-related by a mirror and all the other atoms lie on the mirror. No other abnormal bond lengths and angles deserve discussion.

By a combination of N—H···O and C—H···O hydrogen bonds (Table 1), the ions in (I) are linked into a three-dimensional network. Except for above mentioned, no other interactions, such as π–π, C—H···π*etc*., have been observed.

### Experimental

Acetylacetone (3 ml, 0.03 mol), 3-nitroaniline (4.14 g, 0.03 mol) and a catalytic amount of *p*-toluenesulfonic acid were dissolved in toluene (30 ml). The mixture was refluxed for 6 h and the water was separated azeotropically in a Dean–Stark apparatus. The solvent was removed and the product purified by recrystallization from hexane to yield 4-(3-nitrophenylamino)-3-penten-2-one in 80% yield. To a chloroform (5 ml) solution of the ligand (50 mg, 0.23 mmol) was added triethylamine (0.32 ml, 0.23 mmol) and copper sulfate (37 mg, 0.23 mmol) dissolved in ethanol (25 ml). The resulting brown mixture was filtered and the solution set aside for several days to allow for the formation of colourless blocks of (I); copper was not incorporated into the final product. CH&N elemental analysis calculated for C_12_H_15_N_4_O_8_S: C 38.40, H 4.03, N 14.39%; found: C 38.52, H 4.01, N 14.26%.

### Refinement

H atoms bonded to carbon atoms were placed in idealised positions with C–H = 0.93Å and refined as riding with *U*_iso_(H) = 1.2*U*_eq_(C). Hydrogen atoms bonded to N1 and N3 were firstly found from the difference maps and refined with the constraint of N—H = 0.86 (2)Å and *U*_iso_(H) = 1.2*U*_eq_(N).

### Crystal data

**Table:**

2C_6_H_7_N_2_O_2_^+^·SO_4_^2^^−^	*F*(000) = 776
*M**_r_* = 374.33	*D*_x_ = 1.593 Mg m^−^^3^
Orthorhombic, *P**b**c**m*	Mo *K*α radiation, λ = 0.71073 Å
Hall symbol: -P 2c 2b	Cell parameters from 6890 reflections
*a* = 7.9177 (16) Å	θ = 2.6–28.2°
*b* = 30.843 (6) Å	µ = 0.26 mm^−^^1^
*c* = 6.3924 (13) Å	*T* = 290 K
*V* = 1561.1 (5) Å^3^	Block, colorless
*Z* = 4	0.12 × 0.10 × 0.08 mm

### Data collection

**Table:**

Bruker SMART CCD diffractometer	1845 independent reflections
Radiation source: fine-focus sealed tube	1735 reflections with *I* > 2σ(*I*)
graphite	*R*_int_ = 0.027
ω scans	θ_max_ = 27.0°, θ_min_ = 2.6°
Absorption correction: multi-scan (*SADABS*; Sheldrick, 1997)	*h* = −10→10
*T*_min_ = 0.959, *T*_max_ = 0.979	*k* = −39→38
12364 measured reflections	*l* = −8→8

### Refinement

**Table:**

Refinement on *F*^2^	Primary atom site location: structure-invariant direct methods
Least-squares matrix: full	Secondary atom site location: difference Fourier map
*R*[*F*^2^ > 2σ(*F*^2^)] = 0.061	Hydrogen site location: inferred from neighbouring sites
*wR*(*F*^2^) = 0.144	H-atom parameters constrained
*S* = 1.17	*w* = 1/[σ^2^(*F*_o_^2^) + (0.0402*P*)^2^ + 2.0956*P*] where *P* = (*F*_o_^2^ + 2*F*_c_^2^)/3
1845 reflections	(Δ/σ)_max_ < 0.001
148 parameters	Δρ_max_ = 0.36 e Å^−^^3^
0 restraints	Δρ_min_ = −0.44 e Å^−^^3^

### Special details

**Table:**

Geometry. All e.s.d.'s (except the e.s.d. in the dihedral angle between two l.s. planes) are estimated using the full covariance matrix. The cell e.s.d.'s are taken into account individually in the estimation of e.s.d.'s in distances, angles and torsion angles; correlations between e.s.d.'s in cell parameters are only used when they are defined by crystal symmetry. An approximate (isotropic) treatment of cell e.s.d.'s is used for estimating e.s.d.'s involving l.s. planes.
Refinement. Refinement of *F*^2^ against ALL reflections. The weighted *R*-factor *wR* and goodness of fit *S* are based on *F*^2^, conventional *R*-factors *R* are based on *F*, with *F* set to zero for negative *F*^2^. The threshold expression of *F*^2^ > σ(*F*^2^) is used only for calculating *R*-factors(gt) *etc*. and is not relevant to the choice of reflections for refinement. *R*-factors based on *F*^2^ are statistically about twice as large as those based on *F*, and *R*- factors based on ALL data will be even larger.

### Fractional atomic coordinates and isotropic or equivalent isotropic displacement parameters (Å^2^)

**Table:**

	*x*	*y*	*z*	*U*_iso_*/*U*_eq_	
C1	0.6618 (4)	0.09205 (10)	0.2500	0.0299 (7)	
C2	0.5199 (4)	0.11814 (12)	0.2500	0.0356 (8)	
H2	0.4126	0.1059	0.2500	0.043*	
C3	0.5394 (5)	0.16246 (12)	0.2500	0.0448 (10)	
H3	0.4444	0.1802	0.2500	0.054*	
C4	0.6984 (5)	0.18093 (11)	0.2500	0.0434 (9)	
H4	0.7120	0.2109	0.2500	0.052*	
C5	0.8355 (4)	0.15378 (11)	0.2500	0.0344 (8)	
C6	0.8229 (4)	0.10938 (11)	0.2500	0.0321 (7)	
H6	0.9182	0.0918	0.2500	0.039*	
N1	0.6439 (4)	0.04502 (9)	0.2500	0.0467 (9)	
H1A	0.7456	0.0327	0.2500	0.056*	
H1B	0.5874	0.0368	0.1363	0.056*	
N2	1.0069 (4)	0.17275 (11)	0.2500	0.0446 (8)	
O1	1.1266 (3)	0.14857 (10)	0.2500	0.0581 (9)	
O2	1.0177 (4)	0.21214 (10)	0.2500	0.0713 (11)	
C7	0.1526 (4)	0.10811 (11)	0.7500	0.0313 (7)	
C8	0.0172 (5)	0.13591 (12)	0.7500	0.0376 (8)	
H8	−0.0922	0.1250	0.7500	0.045*	
C9	0.0437 (5)	0.18007 (13)	0.7500	0.0470 (10)	
H9	−0.0482	0.1989	0.7500	0.056*	
C10	0.2057 (5)	0.19658 (12)	0.7500	0.0472 (10)	
H10	0.2246	0.2263	0.7500	0.057*	
C11	0.3388 (4)	0.16774 (12)	0.7500	0.0392 (9)	
C12	0.3170 (4)	0.12348 (11)	0.7500	0.0346 (8)	
H12	0.4088	0.1047	0.7500	0.042*	
N3	0.1249 (4)	0.06160 (9)	0.7500	0.0367 (7)	
H3A	0.0142	0.0558	0.7500	0.044*	
H3B	0.1712	0.0502	0.6268	0.044*	
N4	0.5129 (5)	0.18455 (13)	0.7500	0.0555 (10)	
O3	0.6279 (4)	0.15917 (12)	0.7500	0.0699 (10)	
O4	0.5318 (5)	0.22352 (11)	0.7500	0.0996 (16)	
S1	0.21460 (11)	0.00780 (3)	0.2500	0.0338 (3)	
O5	0.3083 (3)	0.02283 (8)	0.0650 (4)	0.0644 (7)	
O6	0.0436 (4)	0.02484 (9)	0.2500	0.0573 (9)	
O7	0.2120 (4)	−0.03918 (9)	0.2500	0.0680 (11)	

### Atomic displacement parameters (Å^2^)

**Table:**

	*U*^11^	*U*^22^	*U*^33^	*U*^12^	*U*^13^	*U*^23^
C1	0.0313 (16)	0.0263 (15)	0.0322 (17)	−0.0005 (12)	0.000	0.000
C2	0.0252 (16)	0.0411 (19)	0.041 (2)	−0.0013 (14)	0.000	0.000
C3	0.0329 (19)	0.039 (2)	0.063 (3)	0.0096 (15)	0.000	0.000
C4	0.042 (2)	0.0265 (16)	0.061 (3)	0.0013 (15)	0.000	0.000
C5	0.0283 (17)	0.0353 (18)	0.040 (2)	−0.0065 (13)	0.000	0.000
C6	0.0250 (15)	0.0310 (16)	0.0405 (19)	0.0042 (12)	0.000	0.000
N1	0.0395 (17)	0.0301 (15)	0.071 (2)	−0.0051 (13)	0.000	0.000
N2	0.0356 (17)	0.0482 (19)	0.050 (2)	−0.0131 (14)	0.000	0.000
O1	0.0284 (14)	0.069 (2)	0.077 (2)	−0.0047 (13)	0.000	0.000
O2	0.062 (2)	0.0448 (17)	0.107 (3)	−0.0264 (15)	0.000	0.000
C7	0.0336 (17)	0.0297 (16)	0.0305 (17)	−0.0010 (13)	0.000	0.000
C8	0.0302 (17)	0.0420 (19)	0.041 (2)	−0.0013 (14)	0.000	0.000
C9	0.039 (2)	0.040 (2)	0.061 (3)	0.0097 (16)	0.000	0.000
C10	0.052 (2)	0.0294 (18)	0.060 (3)	−0.0031 (16)	0.000	0.000
C11	0.0315 (18)	0.042 (2)	0.044 (2)	−0.0084 (15)	0.000	0.000
C12	0.0292 (16)	0.0328 (17)	0.042 (2)	0.0026 (13)	0.000	0.000
N3	0.0402 (16)	0.0315 (15)	0.0384 (17)	−0.0045 (12)	0.000	0.000
N4	0.043 (2)	0.060 (2)	0.063 (2)	−0.0206 (18)	0.000	0.000
O3	0.0298 (15)	0.094 (3)	0.086 (3)	−0.0086 (16)	0.000	0.000
O4	0.074 (3)	0.057 (2)	0.168 (5)	−0.0364 (19)	0.000	0.000
S1	0.0348 (5)	0.0278 (4)	0.0386 (5)	−0.0022 (3)	0.000	0.000
O5	0.0498 (12)	0.0914 (16)	0.0522 (14)	−0.0095 (11)	0.0027 (11)	0.0215 (12)
O6	0.0408 (16)	0.0383 (14)	0.093 (3)	0.0055 (12)	0.000	0.000
O7	0.0454 (16)	0.0304 (14)	0.128 (3)	−0.0001 (12)	0.000	0.000

### Geometric parameters (Å, °)

**Table:**

C1—C2	1.381 (5)	C7—N3	1.451 (4)
C1—C6	1.383 (4)	C8—C9	1.378 (5)
C1—N1	1.458 (4)	C8—H8	0.9300
C2—C3	1.376 (5)	C9—C10	1.380 (6)
C2—H2	0.9300	C9—H9	0.9300
C3—C4	1.382 (5)	C10—C11	1.379 (5)
C3—H3	0.9300	C10—H10	0.9300
C4—C5	1.371 (5)	C11—C12	1.376 (5)
C4—H4	0.9300	C11—N4	1.472 (5)
C5—C6	1.373 (5)	C12—H12	0.9300
C5—N2	1.478 (4)	N3—H3A	0.8942
C6—H6	0.9300	N3—H3B	0.9368
N1—H1A	0.8900	N4—O3	1.201 (5)
N1—H1B	0.8900	N4—O4	1.211 (5)
N2—O1	1.206 (4)	S1—O7	1.449 (3)
N2—O2	1.218 (4)	S1—O6	1.452 (3)
C7—C8	1.373 (5)	S1—O5^i^	1.471 (2)
C7—C12	1.386 (5)	S1—O5	1.471 (2)
			
C2—C1—C6	121.6 (3)	C7—C8—C9	119.9 (3)
C2—C1—N1	120.0 (3)	C7—C8—H8	120.0
C6—C1—N1	118.3 (3)	C9—C8—H8	120.0
C3—C2—C1	119.2 (3)	C8—C9—C10	120.4 (3)
C3—C2—H2	120.4	C8—C9—H9	119.8
C1—C2—H2	120.4	C10—C9—H9	119.8
C2—C3—C4	120.8 (3)	C11—C10—C9	118.2 (3)
C2—C3—H3	119.6	C11—C10—H10	120.9
C4—C3—H3	119.6	C9—C10—H10	120.9
C5—C4—C3	118.0 (3)	C12—C11—C10	123.0 (3)
C5—C4—H4	121.0	C12—C11—N4	117.8 (3)
C3—C4—H4	121.0	C10—C11—N4	119.2 (3)
C4—C5—C6	123.5 (3)	C11—C12—C7	117.2 (3)
C4—C5—N2	119.0 (3)	C11—C12—H12	121.4
C6—C5—N2	117.5 (3)	C7—C12—H12	121.4
C5—C6—C1	116.9 (3)	C7—N3—H3A	110.2
C5—C6—H6	121.5	C7—N3—H3B	108.1
C1—C6—H6	121.5	H3A—N3—H3B	108.0
C1—N1—H1A	109.6	O3—N4—O4	123.6 (4)
C1—N1—H1B	109.4	O3—N4—C11	118.7 (4)
H1A—N1—H1B	109.5	O4—N4—C11	117.7 (4)
O1—N2—O2	124.2 (3)	O7—S1—O6	110.40 (17)
O1—N2—C5	118.5 (3)	O7—S1—O5^i^	108.80 (12)
O2—N2—C5	117.4 (3)	O6—S1—O5^i^	110.87 (11)
C8—C7—C12	121.3 (3)	O7—S1—O5	108.80 (12)
C8—C7—N3	120.0 (3)	O6—S1—O5	110.87 (11)
C12—C7—N3	118.7 (3)	O5^i^—S1—O5	107.01 (19)
			
C6—C1—C2—C3	0.0	C12—C7—C8—C9	0.0
N1—C1—C2—C3	180.0	N3—C7—C8—C9	180.0
C1—C2—C3—C4	0.0	C7—C8—C9—C10	0.0
C2—C3—C4—C5	0.0	C8—C9—C10—C11	0.0
C3—C4—C5—C6	0.0	C9—C10—C11—C12	0.0
C3—C4—C5—N2	180.0	C9—C10—C11—N4	180.0
C4—C5—C6—C1	0.0	C10—C11—C12—C7	0.0
N2—C5—C6—C1	180.0	N4—C11—C12—C7	180.0
C2—C1—C6—C5	0.0	C8—C7—C12—C11	0.0
N1—C1—C6—C5	180.0	N3—C7—C12—C11	180.0
C4—C5—N2—O1	180.0	C12—C11—N4—O3	0.0
C6—C5—N2—O1	0.0	C10—C11—N4—O3	180.0
C4—C5—N2—O2	0.0	C12—C11—N4—O4	180.0
C6—C5—N2—O2	180.0	C10—C11—N4—O4	0.0

Symmetry codes: (i) *x*, *y*, −*z*+1/2.

### Hydrogen-bond geometry (Å, °)

**Table:**

*D*—H···*A*	*D*—H	H···*A*	*D*···*A*	*D*—H···*A*
N1—H1A···O6^ii^	0.89	2.37	3.226 (4)	161
N1—H1B···O5	0.89	2.30	2.987 (4)	134
N1—H1B···O5^iii^	0.89	2.39	2.928 (3)	119
N3—H3A···O7^iv^	0.89	1.86	2.755 (4)	176
N3—H3B···O5^i^	0.94	1.84	2.756 (3)	164
C4—H4···O4^v^	0.93	2.48	3.229 (5)	138
C6—H6···O6^ii^	0.93	2.29	3.139 (4)	151
C8—H8···O3^vi^	0.93	2.45	3.165 (5)	133
C10—H10···O2^vii^	0.93	2.51	3.184 (5)	130

Symmetry codes: (ii) *x*+1, *y*, *z*; (iii) −*x*+1, −*y*, −*z*; (iv) −*x*, −*y*, −*z*+1; (i) *x*, *y*, −*z*+1/2; (v) *x*, −*y*+1/2, −*z*+1; (vi) *x*−1, *y*, *z*; (vii) *x*−1, −*y*+1/2, −*z*+1.
